# Dual-energy CT angiography in suspected pulmonary embolism: influence of injection protocols on image quality and perfused blood volume

**DOI:** 10.1007/s10554-020-01911-8

**Published:** 2020-06-06

**Authors:** Aleksander Kosmala, Philipp Gruschwitz, Simon Veldhoen, Andreas Max Weng, Bernhard Krauss, Thorsten Alexander Bley, Bernhard Petritsch

**Affiliations:** 1 Department of Diagnostic and Interventional Radiology, University Hospital Würzburg, Oberdürrbacher Straße 6, 97080 Würzburg, Germany; 2 Siemens Healthcare GmbH, Research and Development, Forchheim, Germany

**Keywords:** CT, Dual-energy CT, Pulmonary embolism, Contrast media

## Abstract

To compare intravenous contrast material (CM) injection protocols for dual-energy CT pulmonary angiography (CTPA) in patients with suspected acute pulmonary embolism with regard to image quality and pulmonary perfused blood volume (PBV) values. A total of 198 studies performed with four CM injection protocols varying in CM volume and iodine delivery rates (IDR) were retrospectively included: (A) 60 ml at 5 ml/s (IDR = 1.75gI/s), (B) 50 ml at 5 ml/s (IDR = 1.75gI/s), (C) 50 ml at 4 ml/s (IDR = 1.40gI/s), (D) 40 ml at 3 ml/s (IDR = 1.05gI/s). Image quality and PBV values at different resolution settings were compared. Pulmonary arterial tract attenuation was highest for protocol A (397 ± 110 HU; *p* vs. B = 0.13; vs. C = 0.02; vs. D < 0.001). CTPA image quality of protocol A was rated superior compared to protocols B and D by reader 1 (*p* = 0.01; < 0.001), and superior to protocols B, C and D by reader 2 (*p* < 0.001; 0.02; < 0.001). Otherwise, there were no significant differences in CTPA quality ratings. Subjective iodine map ratings did not vary significantly between protocols A, B, and C. Both readers rated protocol D inferior to all other protocols (*p* < 0.05). PBV values did not vary significantly between protocols A and B at resolution settings of 1, 4 and 10 (*p* = 0.10; 0.10; 0.09), while otherwise PBV values displayed a decreasing trend from protocol A to D (*p* < 0.05). Higher CM volume and IDR are associated with superior CTPA and iodine map quality and higher absolute PBV values.

## Introduction

Pulmonary embolism (PE) is a frequent cardiovascular disease with potentially life-threatening acute and chronic complications [1]. For a fast and accurate diagnosis, computed tomography pulmonary angiography (CTPA) has replaced conventional pulmonary angiography and V/Q-scans [2–4]. CTPA visualizes the embolus itself, yet provides no further information about its functional effects on pulmonary perfusion, which might be of higher pathophysiological importance [5].

The introduction of dual-energy (DE) CT provides additional functional data at similar or lower radiation exposure compared to standard single energy CTPA [5–11]. By simultaneous acquisition of two CTPA datasets at different tube energies, the energy dependent X-ray attenuation characteristics of certain elements like calcium and iodine can be utilized for material quantification [12, 13]. The use of postprocessing software allows for direct visualization of pulmonary iodine distribution maps, which can be analyzed for perfusion defects, thus improving diagnostic performance of CTPA [14–18]. Moreover, the automatic quantification of pulmonary perfused blood volume (PBV) enables fast and reader-independent evaluation iodine distribution within the lung parenchyma, which correlates with thrombus load, laboratory parameters of PE severity, and admission to an intensive care unit [10, 15, 17, 19–21].

As the pulmonary iodine content is derived from a single-timepoint scan, PBV values are likely to depend on the timing and shape of the contrast bolus. To optimally combine both, the high-resolution morphological information from CTPA, and the functional information derived from DE data, it is necessary to adapt the contrast material (CM) injection protocol. CTPA requires maximum contrast within the pulmonary arteries, whereas adequate attenuation of the downstream pulmonary capillary system is needed for PBV evaluation and homogeneous iodine maps. Additionally, attenuation within the venous inflow tract should be minimized to reduce the occurrence of artifacts resulting from highly concentrated CM, which can impair correct assessment of iodine maps [22–24]. Despite the apparent pivotal role of an optimized CM injection protocol for DE-CTPA, reports on CM injection protocol optimization are available based on first-generation dual-source DE scanners only [25–27]. Moreover, there is a clinical need to investigate the effects of different CM injection protocols on PBV values.

Therefore, the purpose of this study was to compare four CM injection protocols for DE-CTPA on a latest (third) generation dual-source scanner in patients with suspected acute PE with regard to image quality and absolute PBV values.

## Materials and methods

### Patients

**Fig. 1:**
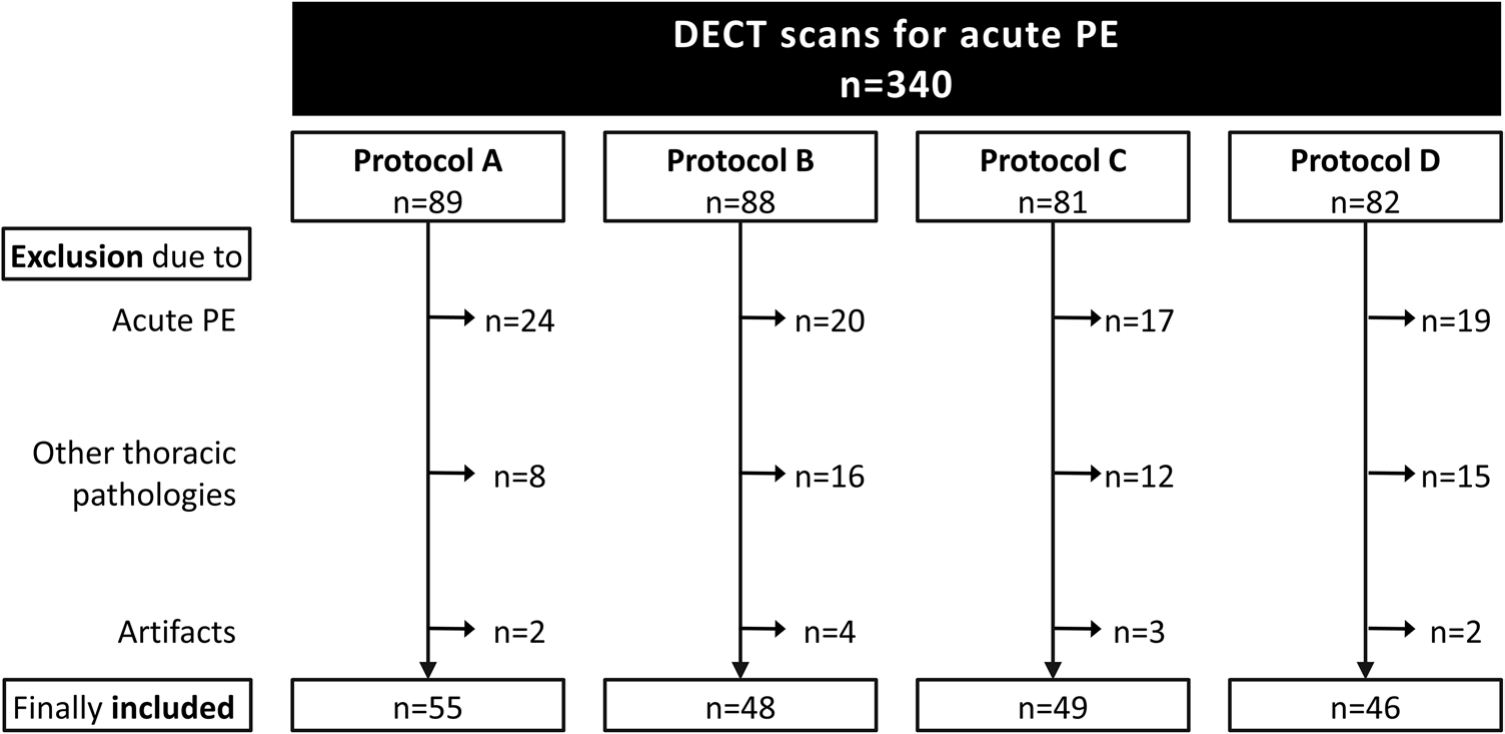
Flowchart demonstrating selection of studies. *DECT* dual-energy computed tomography, *PE* pulmonary embolism

The ethics committee waived the need for informed patient consent for this retrospective analysis. We evaluated data of patients who had undergone DE-CTPA for clinically suspected acute PE at our institution within a 20 months period. The CM injection protocol for DE-CTPA underwent 3 modifications during this period. In total, 340 eligible studies were identified. Then, studies with a positive diagnosis of PE, significant artifacts or significant pulmonary abnormalities that might hamper correct PBV analysis (e.g. atelectasis, pneumonia, emphysema, known bronchiolitis or chronic airway disease) [15] were excluded, leaving a total of 198 scans for inclusion into this study (Fig. 1).

### Scanner settings and contrast injection protocols

All examinations were performed on a 3rd-generation dual-source CT scanner (Somatom Force, Siemens Healthcare GmbH) in dual-energy technique using identical scanner settings (collimation, 196 × 0.6 mm; pitch, 0.55; rotation time, 0.25 s). Tube voltages were set to 90 kV (tube A) and 150 kV (tube B, with activated tin filter). Automatic tube current modulation (CARE Dose 4D; Siemens) was used in all patients, with the reference tube current time product set to 60/46 mAs (tube A/B). All scans were acquired in mid-inspiratory breath hold in caudocranial direction.

An automated injector (CT motion, Ulrich-GmbH) was used to administer pre-warmed iodinated contrast medium with 350 mgI/mL (Imeron 350, Bracco) at varying flow rates/iodine delivery rates (IDR) and volumes (Table 1) via an 18 Gauge cubital vein catheter: protocol A consisted of 60 ml CM injected at a flow rate of 5 ml/s, protocol B 50 ml at a flow rate of 5 ml/s, protocol C 50 ml at 4 ml/s, and protocol D 40 ml at 3 ml/s. All CM injections were followed by a 50 ml saline chaser administered with an identical flow rate to the CM. Bolus tracking was initiated 5 s after CM injection start, based on a ROI within the pulmonary trunk. The triggering threshold was set to 120 HU above baseline, with a scan delay of 7 s.

**Table 1:** Contrast material injection protocols, patient demographics and radiation dose output

Protocol	A	B	C	D
Total CM volume (mL)	60	50	50	40
Injection rate (mL/s)	5	5	4	3
Iodine delivery rate (gI/s)	1.75	1.75	1.40	1.05
Injection time (s)	12	10	12.5	13.3
Total iodine load (g)	21	17.5	17.5	14
Number of patients	55	48	49	46
Male/female	26/29	17/31	25/24	25/21
Age (years)	65 ± 17	66 ± 18	65 ± 16	60 ± 18
CTDI_vol_ (mGy)	3.8	3.9	3.9	3.7
DLP (mGy*cm)	139.4	138.9	138.3	136.6

*CM* contrast material, *I* Iodine, *CTDI*_*vol*_ volumetric computed tomography dose index, *DLP* dose length product

### Image reconstruction and analysis

Three CTPA image stacks were reconstructed at 2.0 mm thickness using a medium soft kernel (QR40) and activated iterative beam-hardening correction (iBHC): 150 kV images, 90 kV images and weighted average images with imaging characteristics similar to a single-energy 120 kV scan.

Additionally, iodine distribution maps were generated using commercially available software (Lung PBV, Syngo version VA30A, Siemens). Based on a three material decomposition algorithm (soft tissue, air, iodine), the software is able to quantify the iodine content within a voxel and generate color-coded iodine distribution maps of the lung [13]. These iodine distribution images have been shown to be a reliable surrogate for pulmonary parenchymal perfusion [20]. For the purpose of this study, we evaluated absolute parenchymal enhancement in HU based on pulmonary perfused blood volume (PBV) attenuation values for the whole lung, provided by the software after automatic lung segmentation. Furthermore, we analyzed three different resolution settings for iodine map visualization: 1, 4 (predefined standard value) and 10 on a scale from 1 to 10. These resolution settings affect the range of the smoothing filter, with low values leading to improved spatial resolution, and high values to improved image contrast. It is expected that quantum statistics will be important at low resolution values, while systematic deviations should dominate at high resolution values. For each resolution, color-coded iodine maps with a slice thickness of 3.0 mm were reconstructed.

For quantitative evaluation of CTPA images, circular ROIs were placed on weighted average images in transversal orientation at identical anatomic locations for each patient: the subclavian vein on the side of CM administration, superior vena cava, pulmonary trunk, right lower lobe pulmonary artery, left upper lobe pulmonary artery, descending aorta, the latissimus dorsi muscle and subcutaneous fat at the level of the pulmonary trunk. ROIs were drawn as large as possible, while excluding vessel walls, adjacent structures and any areas of obvious heterogeneity (e.g. artifacts). Mean attenuation values and standard deviations (SDs) were recorded for each ROI. In order to minimize inaccuracies and provide concise results, attenuation measurements were then combined into three groups: the venous tract, the pulmonary arterial tract and the aortic tract. The venous tract is composed of the subclavian vein and the superior vena cava, the pulmonary arterial tract consists of the pulmonary trunk and pulmonary arteries, the aortic tract includes the descending aorta. Signal-to-noise ratios (SNR) and contrast-to-noise ratios (CNR) were calculated as described before [7, 28]:

$$SNR = \frac{{Mean \;attenuation_{vessel} }}{{SD_{vessel} }}$$

$$CNR = \frac{{\left( {Mean\; attenuation_{vessel} - Mean\; attenuation_{muscle} } \right) }}{{SD_{fat} }}$$

Two independent readers (BP and AK, with 11 and 6 years of experience in cardiovascular radiology) blinded to the injection protocol, scan date and resolution setting evaluated the subjective image quality of weighted average CTPA images and three sets of color-coded iodine maps for each patient on a 5-point scale (1 = excellent image quality; 2 = good image quality; 3 = moderate image quality; 4 = fair image quality 5 = poor image quality).

### Statistical analysis

Statistical analyses were performed using dedicated software (SPSS Statistics version 25; IBM). Continuous variables are displayed as means ± SD and compared via the Mann–Whitney U test. Interrater agreement was determined by calculating the absolute percentage of agreement between the readers and ratings are presented in percentages per group and rating. Ratings for different groups were compared via parametric testing as suggested by Sullivan and Artino [29]. *p*-values ≤ 0.05 were considered statistically significant.

## Results

In all 198 included studies there were no periprocedural complications or non-diagnostic CTPAs. Demographic data of included patients is summarized in Table 1. Specific patient characteristics like body weight, body mass index or heart rate were not recorded at our institution during the specified timeframe.

### Objective image quality

**Fig. 2:**
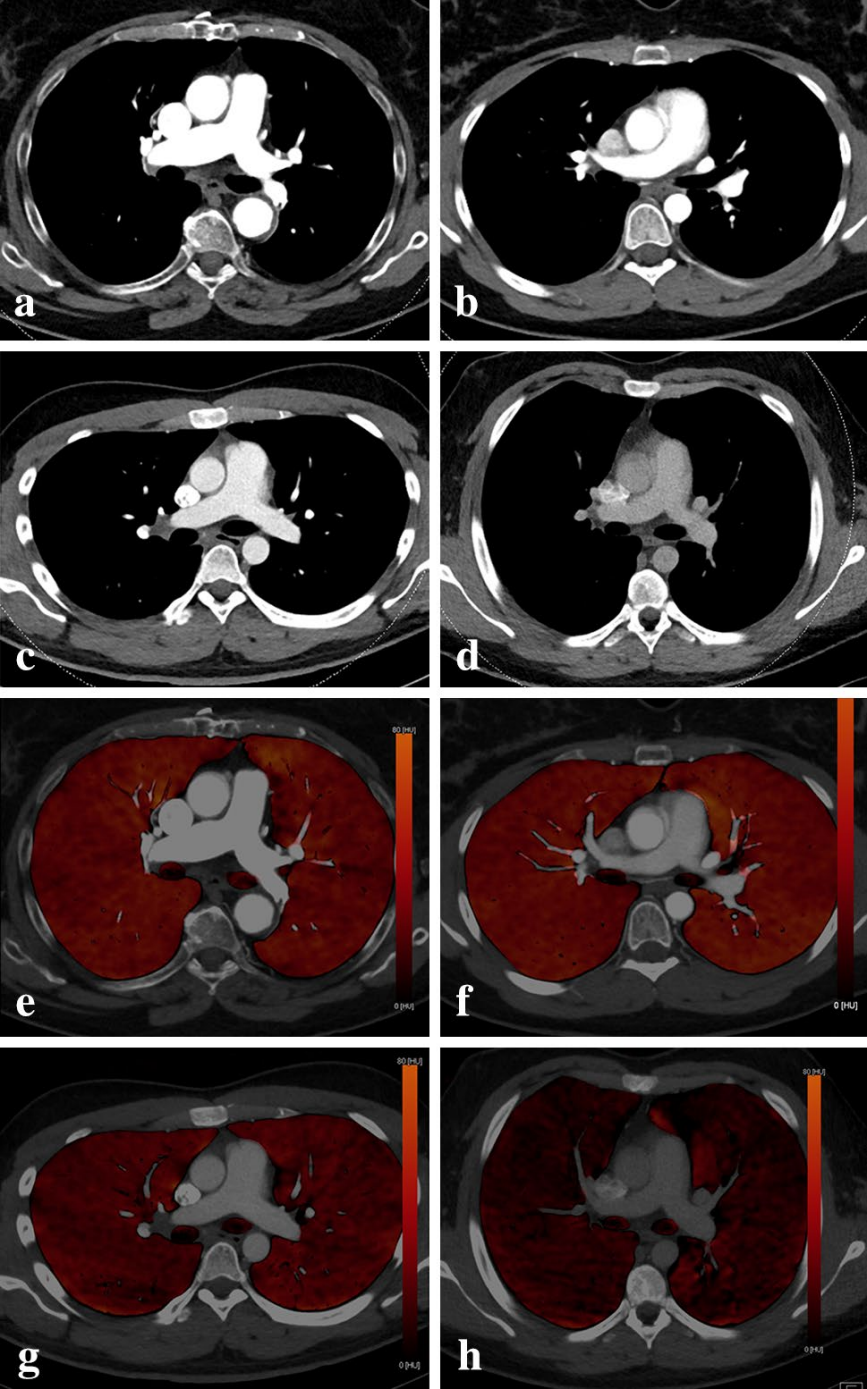
CT pulmonary angiography (**a**–**d**) and iodine map (**e**–**h**) with identical window settings for protocol A (**a**, **e**), B (**b**, **f**), C (**c**, **g**) and D (**d**, **h**). Note the decrease in pulmonary vascular contrast and iodine map enhancement from A to D

Key findings for objective image quality are shown in Table 2, with corresponding p-values listed in Table 3. Enhancement in the pulmonary arterial tract and aortic tract was lowest in protocol D, where the least CM was administered, and highest in protocol A, where the most CM was injected. However, there were no significant differences in average pulmonary arterial attenuation between the two high-flow protocols (A and B with 5 ml/s), and between the two protocols with intermediate contrast volume (B and C with 50 ml). Protocol D showed significantly lower SNR and CNR-values in the pulmonary arterial tract compared to all other protocols, while no significant differences were observed between protocols A, B and C concerning pulmonary arterial SNR or CNR. For the aortic tract, there was no significant difference in attenuation and SNR between protocols A and B, while aortic enhancement and SNR decreased significantly with lower flow or lower CM volume in protocols C and D. Aortic tract CNR was significantly lower for protocol D compared to all other protocols, whereas protocols A, B and C showed no significant differences. Venous tract attenuation was highest for protocol D, and lowest for protocol B, with intermediate values in protocols A and C. Figure 2 demonstrates exemplary CTPA and iodine map images for each protocol.

**Table 2:** Objective image quality. Attenuation, signal-to-noise ratio (SNR), and contrast-to-noise ratio (CNR) for the venous tract (subclavian vein + superior vena cava), pulmonary arterial tract (main pulmonary artery + right lower lobe artery + left upper lobe artery), and aortic tract (descending aorta) for all four protocols

	Protocol A	Protocol B	Protocol C	Protocol D
Attenuation [HU]				
Venous tract	597 ± 275	367 ± 207	690 ± 381	1028 ± 486
Pulmonary arterial tract	397 ± 110	369 ± 145	343 ± 110	285 ± 85
Aortic tract	289 ± 84	283 ± 135	215 ± 63	144 ± 41
SNR				
Venous tract	8.6 ± 4.8	10.4 ± 4.9	6.6 ± 4.2	6.0 ± 3.9
Pulmonary arterial tract	24.8 ± 6.3	23.8 ± 7.9	24.3 ± 7.7	19.1 ± 5.1
Aortic tract	20.3 ± 7.8	21.5 ± 9.9	17.2 ± 5.9	11.1 ± 4.1
CNR				
Venous tract	54.0 ± 27.9	35.0 ± 22.5	70.1 ± 41.0	92.6 ± 58.7
Pulmonary arterial tract	34.6 ± 13.7	35.2 ± 16.4	34.8 ± 15.6	23.8 ± 9.4
Aortic tract	24.0 ± 9.8	26.1 ± 15.1	20.2 ± 9.6	9.9 ± 5.3
Whole lung PBV-values [HU]				
Resolution 1	32.6 ± 8.4	30.7 ± 10.2	23.5 ± 6.3	17.7 ± 5.2
Resolution 4	32.7 ± 8.5	30.8 ± 10.3	23.6 ± 6.3	17.6 ± 5.1
Resolution 10	32.8 ± 8.5	30.8 ± 10.4	23.7 ± 6.4	17.5 ± 5.2

Pulmonary perfused blood volume (PBV) HU values for the whole lung are provided as measure of parenchymal enhancement. All values shown as means ± standard deviation

**Table 3:** The p-values for pairwise comparisons of objective image quality markers shown in Table 2

	A vs B	A vs C	A vs D	B vs C	B vs D	C vs D
Attenuation						
Venous tract	** < 0.001**	0.435	** < 0.001**	** < 0.001**	** < 0.001**	**0.001**
Pulmonary arterial tract	0.130	**0.023**	** < 0.001**	0.665	**0.003**	**0.006**
Aortic tract	0.597	** < 0.001**	** < 0.001**	** < 0.001**	** < 0.001**	** < 0.001**
SNR						
Venous tract	**0.037**	**0.004**	** < 0.001**	** < 0.001**	** < 0.001**	0.622
Pulmonary arterial tract	0.376	0.484	** < 0.001**	0.784	**0.002**	**0.001**
Aortic tract	0.418	**0.037**	** < 0.001**	**0.011**	** < 0.001**	** < 0.001**
CNR						
Venous tract	** < 0.001**	**0.044**	** < 0.001**	** < 0.001**	** < 0.001**	**0.028**
Pulmonary arterial tract	0.932	0.858	** < 0.001**	0.840	** < 0.001**	** < 0.001**
Aortic tract	0.517	0.065	** < 0.001**	0.065	** < 0.001**	** < 0.001**
Whole lung PBV-attenuation						
Resolution 1	0.101	** < 0.001**	** < 0.001**	** < 0.001**	** < 0.001**	** < 0.001**
Resolution 4	0.096	** < 0.001**	** < 0.001**	**0.001**	** < 0.001**	** < 0.001**
Resolution 10	0.090	** < 0.001**	** < 0.001**	**0.001**	** < 0.001**	** < 0.001**

Mean attenuation, *SNR* signal-to-noise ratio, *CNR* contrast-to-noise ratio, *PBV* whole lung perfused blood volume. Significant differences are shown in bold text

**Fig. 3:**
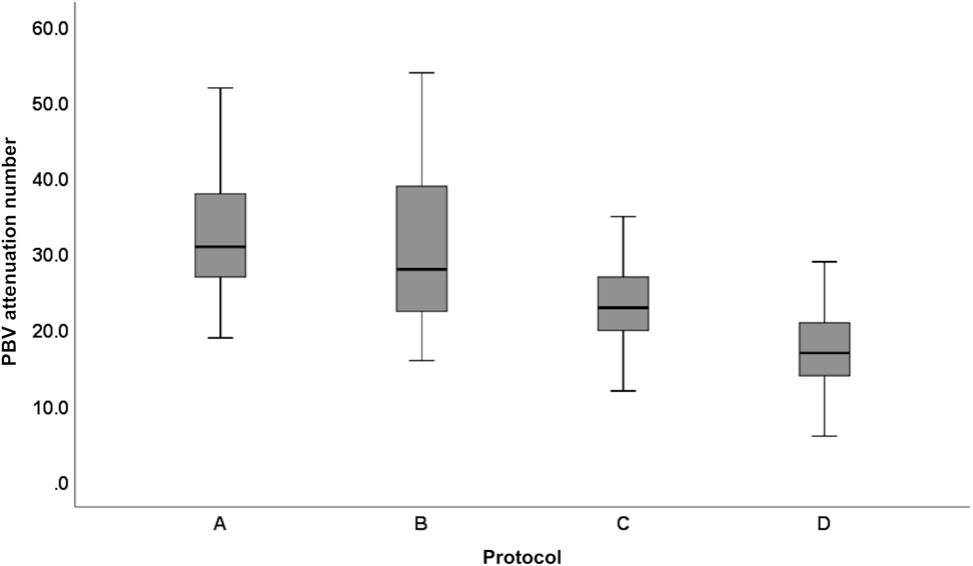
Boxplot of pulmonary perfused blood volume (PBV) attenuation numbers for protocol A, B, C, and D at a resolution setting of 4

A comprehensive overview of whole lung PBV attenuation values is displayed in Fig. 3. For all resolution settings, we found no significant difference between PBV values of protocols A and B. All other pairwise protocol-comparisons revealed significant differences between PBV values, with PBV values decreasing from protocol A to protocol D (Tables 2 and 3, Fig. 3).

### Subjective image quality

**Fig. 4:**
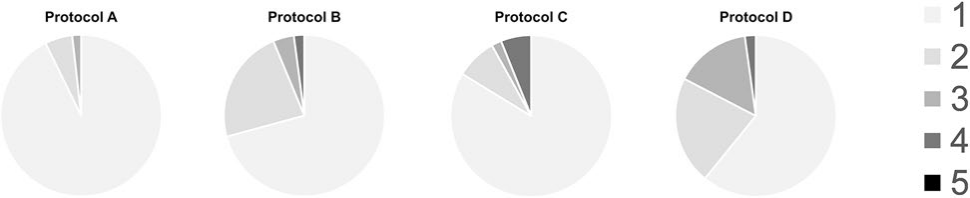
Pie charts of computed tomography pulmonary angiography (CTPA) subjective image quality ratings of reader 2 (from 1 = excellent image quality, no artifacts and superior diagnostic confidence, to 5 = non-diagnostic image)

A complete summary of subjective image quality ratings of CTPA images and iodine maps is provided in Table 4. Overall, readers showed an excellent interobserver agreement, with a percentage of agreement of 0.92, 0.88, 0.83 and 0.86 for subjective ratings of CTPA and iodine map at resolutions 1, 4, and 10, respectively. Median CTPA image quality rating was 1 for all protocols for both readers, with excellent ratings of 94.5/92.7% (R1/R2) for protocol A, 68.8/70.8% for protocol B, 81.6/83.7% for protocol C, and 67.4/60.9% for protocol D (Fig. 4). Reader 1 rated CTPA image quality significantly better for protocol A compared to protocol B and protocol D (p < 0.05 for both comparisons), while reader 2 rated protocol A superior to all other protocols (p < 0.05 for all comparisons). Otherwise, the protocols displayed no significant differences in pairwise comparisons.

**Table 4:** Subjective image quality ratings of reader 1 (R1) and reader 2 (R2) for computed tomography pulmonary angiographies (CTPA) and iodine maps (resolutions 1, 4, and 10) for all four protocols on a five-point scale

Likert scale	Protocol A		Protocol B		Protocol C		Protocol D	
	R1	R2	R1	R2	R1	R2	R1	R2
CTPA								
1	94.5	92.7	68.8	70.8	81.6	83.7	67.4	60.9
2	5.5	5.5	25.0	22.9	10.2	8.2	10.9	21.7
3	–	1.8	4.2	4.2	4.1	2.0	19.6	15.2
4	–	–	2.1	2.1	4.1	6.1	2.2	2.2
5	–	–	–	–	–	–	–	–
Median	1	1	1	1	1	1	1	1
Iodine map resolution 1								
1	58.2	61.8	62.5	64.6	67.3	69.4	23.9	32.6
2	36.4	32.7	31.3	29.2	20.4	22.4	65.2	45.7
3	1.8	3.6	2.1	4.2	8.2	4.1	8.7	19.6
4	3.6	1.8	4.2	2.1	4.1	4.1	2.2	2.2
5	–	–	–	–	–	–	–	–
Median	1	1	1	1	1	1	2	2
Iodine map resolution 4								
1	56.4	65.5	64.6	64.6	69.4	65.3	23.9	28.3
2	38.2	29.1	31.3	29.2	18.4	28.6	47.8	45.7
3	5.5	5.5	2.1	4.2	8.2	2.0	26.1	21.7
4	–	–	2.1	2.1	4.1	4.1	2.2	4.3
5	–	–	–	–	–	–	–	–
Median	1	1	1	1	1	1	2	2
Iodine map resolution 10								
1	58.2	60.0	62.5	56.3	61.2	57.1	23.9	23.9
2	36.4	30.9	25.0	29.2	18.4	24.5	39.1	37.0
3	5.5	7.3	8.3	10.4	12.2	10.2	23.9	21.7
4	–	1.8	4.2	4.2	8.2	8.2	10.9	15.2
5	–	–	–	–	–	–	2.2	2.2
Median	1	1	1	1	1	1	2	2

Lower scores indicate higher image quality. Values provided as percentages

Subjective quality of iodine maps for protocol D (median 2 for all resolutions) was rated significantly inferior comparted to the other protocols (all median 1 for all resolutions*;* p < 0.05 for all comparisons for both readers). Protocols A, B and C showed no significant differences in pairwise comparisons for all resolutions and both readers. A groupwise comparison of iodine map ratings at resolution settings of 1 and 4 did not result in any significant differences for both readers, while studies reconstructed with a resolution setting of 10 were rated significantly lower by both readers (p < 0.05 for all groupwise comparisons). At the standard resolution level of 4, the iodine map image quality was rated excellent or good in 94.6/94.6% (R1/R2) of scans in protocol A, 95.9/93.8% in protocol B, 87.8/93.9% in protocol C, and 71.7/74.0% in protocol D (Table 4).

## Discussion

We aimed to evaluate image quality and absolute DE PBV values of four different CM injection protocols for DE-CTPA in patients with acute PE. Our results show that for CTPA image quality, high volume of CM injected at high flow rates/IDR yields optimal results for subjective quality as well as pulmonary arterial attenuation, although pulmonary arterial SNR and CNR were comparable between the high- and intermediate-volume/IDR protocols (protocols A, B, and C). We also demonstrate that higher CM volume and IDR are associated with higher absolute PBV values. However, subjective image quality of color-coded iodine maps did not differ significantly between high- and intermediate-volume/IDR protocols.

CTPA is the established imaging method of choice in patients with suspected acute PE due to its high diagnostic accuracy [1, 4]. Performing CTPA in dual-energy technique offers further potential advantages, foremost in the form of iodine maps, which have been shown to correspond well to scintigraphic findings in the acute setting and enhance the detection of peripheral pulmonary arterial clots [5, 14, 18]. Moreover, iodine maps may also have prognostic value superior to the regular CTPA obstruction score [30], as several studies have shown a correlation between the extent of perfusion defects on iodine maps and clinical embolism severity as well as adverse clinical outcome [21, 31, 32].

For ideal performance of DE-CTPA, it is of critical importance that the arrival of the contrast bolus in the pulmonary vasculature and the data acquisition are timed optimally. On one hand, high attenuation of pulmonary arteries is required to depict pulmonary emboli. On the other hand, a sufficient latency between contrast bolus influx and scan is also desirable, as the CM needs sufficient distribution time in the capillary bed to enable optimal assessment of pulmonary “perfusion” via iodine maps or absolute PBV values. Previous studies have demonstrated that CM bolus geometry is dependent on several factors, including CM volume and iodine concentration, IDR and injection rate [26, 33–36]. As we use CM of only one specific concentration at our institute, we chose to manipulate IDR by changing the injection flow rate. Similar to other studies, we found that pulmonary arterial vessel attenuation tended to correlate positively with IDR [26, 27, 33, 37]. However, this only translated into superior CTPA image quality ratings when combined with a high CM volume (protocol A). Also, protocol B with intermediate CM volume and high IDR resulted in a relatively tight bolus of 10 s injection time, which might have hampered robust scan timing over a broad spectrum of patients with variable cardiac output, leading to more scans with suboptimal vessel attenuation. Accordingly, protocol B resulted in fewer “excellent” image quality ratings than protocol C, despite equal total CM volume.

To our knowledge, this is the first study to evaluate the influence of CM injection protocols on absolute PBV values. Our findings indicate that out of IDR, total CM volume and iodine load, a high IDR might be the key factor to obtain higher PBV values: at standard resolution settings, protocol A and B (both high-IDR protocols at 1.75 gI/s) showed no significant differences in PBV at different CM volumes and total iodine loads. On the other hand, when comparing protocols B and C (both with equal CM volume and iodine load), the high-IDR protocol B resulted in significantly higher overall PBV values (30.8 vs. 23.7). However, higher absolute PBV values did not generally translate into superior subjective image quality of iodine maps, as protocols A, B and C received similar subjective image quality ratings. Consequently, apart from high pulmonary parenchymal enhancement expressed by high PBV values, also the ratio of contrast uptake to resulting artefacts has to be taken into account.

Earlier studies performed on previous generations of dual-source scanners have unvaryingly reported the susceptibility of iodine maps to beam hardening artifacts and heart motion, which may potentially lead to false positive diagnoses of “perfusion” defects caused by PEs [5, 24–27, 38]. In our study, however, the use of a third generation dual-source scanner with novel iBHC reconstructions and improved DE postprocessing algorithms allowed for minimization of beam hardening artifacts, while studies with significant artifacts due to motion or metallic implants were excluded. Therefore, we didn’t perform a separate analysis of artifacts.

While previous reports typically relied on iodine loads around 40 g [25, 28] to 30 g [26, 27], at our institution routinely minimal doses of CM with total iodine loads around 20 g and less are used to obtain sufficient image quality and enhancement. In times of increasing numbers of contrast-based CT-scans, we believe that this is an important step to minimize nephrotoxicity associated with iodinated contrast media. Based on our results, we suggest the use of a protocol with moderate to high CM volume/iodine load (21–17.5 g I) and IDR (1.75–1.4 gI/s). We believe that an ideal bolus should be sufficiently fitted to offer high pulmonary vascular attenuation for ideal CTPA evaluation, while at the same time providing adequate pulmonary parenchymal enhancement for homogeneous iodine maps. Therefore, protocol C remained our standard protocol used in daily clinical routine, as it proved to provide the most robust bolus for a broad spectrum of patients, while simultaneously maintaining satisfactory subjective and objective quality of both CTPA and iodine images at a reasonable Iodine exposure.

We acknowledge that there are some limitations in this study. The purpose of the study was to investigate the effect of different CM injection protocols on image quality; hence we did not compare the diagnostic performance of certain protocols for detection of PE and we did not evaluate potential additional benefits of iodine maps for PE detection and clinical management. Although we initially excluded any scans with thoracic pathologies and significant artifacts, it cannot be ruled out that some scans in our study contained minor artifacts in iodine maps. These artifacts may have influenced absolute PBV values. We also emphasize, that for the purpose of this study, we chose to exclude scans with patient-related or external artifacts that might have hampered correct PBV analysis, thus creating groups with an ‘ideal’ image quality, that might not be achieved in ‘real-world’ settings, where generally more robust contrast injection protocol are favored. Results may also have been influenced by alterations in contrast bolus geometry due to cardiac output differences. To avoid this, an intragroup comparison with multiple CTs per patient would have been necessary, which is obviously precluded by ethical reasons. We also recognize that vascular attenuation and PBV values were assessed at a fixed time point for all protocols, while enhancement curves could have provided additional information. Additionally, we acknowledge that differences in patients’ body mass index could have influenced image quality. Furthermore, we used PBV values as surrogate for pulmonary parenchymal perfusion without comparison to a standard of reference, such as ventilation/perfusion scintigraphy, SPECT or MR perfusion imaging. While this would have exceeded the retrospective nature of this study, further research is needed to compare PBV values with perfusion quantified by other imaging modalities. Also, our results might not be applicable for other scanners and vendors. Future studies should also manipulate other CM injection parameters such as iodine concentration or scan delay as well as introduce new split-bolus techniques.

In conclusion, our findings show that higher CM volume and IDR are associated with superior CTPA and iodine map quality as well as higher absolute PBV values. A clinically robust protocol with intermediate CM volume and IDR can be a sensible tradeoff for daily practice, as it provides satisfactory subjective and objective image quality for morphologic and “functional” images at a moderate iodine exposure.

## Data Availability

The corresponding author has full control of all primary data and agrees to allow the journal to review data if requested.

## Abbreviations

CM: Contrast material
CNR: Contrast-to-noise ratio
CTDI_vol_: Volume computed tomography dose index;
CTPA: Computed tomography pulmonary angiography
DE: Dual-energy
DLP: Dose-length product
iBHC: Iterative beam-hardening correction
IDR: Iodine delivery rate
PBV: Perfused blood volume
PE: Pulmonary embolism
SD: Standard deviation
SNR: Signal-to-noise ratio
